# Adherence to pre-set benchmark quality criteria to qualify as expert assessor of dysplasia in Barrett’s esophagus biopsies – towards digital review of Barrett’s esophagus

**DOI:** 10.1177/2050640619853441

**Published:** 2019-05-21

**Authors:** MJ van der Wel, E Klaver, LC Duits, RE Pouw, CA Seldenrijk, GJA Offerhaus, M Visser, FJW ten Kate, K Biermann, LAA Brosens, M Doukas, C Huysentruyt, A Karrenbeld, G Kats-Ugurlu, JS van der Laan, G van Lijnschoten, FCP Moll, AHAG Ooms, JG Tijssen, JJGHM Bergman, SL Meijer

**Affiliations:** 1 Department of Pathology, Amsterdam University Medical Centers, Amsterdam, the Netherlands; 2 Department of Gastroenterology and Hepatology, Amsterdam University Medical Centers, Amsterdam, the Netherlands; 3 Department of Pathology, Pathology-DNA BV, St. Antonius Hospital, Nieuwegein, the Netherlands; 4 Department of Pathology, University Medical Center, Utrecht, The Netherlands; 5 Department of Pathology, Symbiant BV, Zaans Medical Center, Zaandam, the Netherlands; 6 Department of Pathology, Erasmus Medical Center, Rotterdam, The Netherlands; 7 Department of Pathology, Stichting PAMM, Eindhoven, The Netherlands; 8 Department of Pathology, Academic Medical Center Groningen, Groningen, The Netherlands; 9 Department of Pathology, Haga Hospital, The Hague, The Netherlands; 10 Department of Pathology, Isala Clinics, Zwolle, The Netherlands; 11 Department of Pathology, Pathan BV, St. Fransiscus Vlietland Hospital, Rotterdam, the Netherlands; 12 Department of Cardiology, Amsterdam University Medical Centers, Amsterdam, the Netherlands

**Keywords:** Barrett’s esophagus, digital microscopy, whole slide imaging, benchmark quality criteria, consensus gold standard diagnosis, review panel, observer agreement

## Abstract

**Background:**

Dysplasia assessment of Barrett’s esophagus biopsies is associated with low observer agreement; guidelines advise expert review. We have developed a web-based review panel for dysplastic Barrett’s esophagus biopsies.

**Objective:**

The purpose of this study was to test if 10 gastrointestinal pathologists working at Dutch Barrett’s esophagus expert centres met pre-set benchmark scores for quality criteria.

**Methods:**

Ten gastrointestinal pathologists twice assessed 60 digitalized Barrett’s esophagus cases, enriched for dysplasia; then randomised (7520 assessments). We tested predefined benchmark quality criteria: (a) percentage of ‘indefinite for dysplasia’ diagnoses, benchmark score ≤14% for all cases, ≤16% for dysplastic subset, (b) intra-observer agreement; benchmark score ≥0.66/≥0.39, (c) percentage agreement with ‘gold standard diagnosis’; benchmark score ≥82%/≥73%, (d) proportion of cases with high-grade dysplasia underdiagnosed as non-dysplastic Barrett’s esophagus; benchmark score ≤1/78 (≤1.28%) assessments for dysplastic subset.

**Results:**

Gastrointestinal pathologists had seven years’ Barrett’s esophagus-experience, handling seven Barrett’s esophagus-cases weekly. Three met stringent benchmark scores; all cases and dysplastic subset, three met extended benchmark scores. Four pathologists lacked one quality criterion to meet benchmark scores.

**Conclusion:**

Predefined benchmark scores for expert assessment of Barrett’s esophagus dysplasia biopsies are stringent and met by some gastrointestinal pathologists. The majority of assessors however, only showed limited deviation from benchmark scores. We expect further training with group discussions will lead to adherence of all participating gastrointestinal pathologists to quality criteria, and therefore eligible to join the review panel.

## Key summary

- Barrett’s esophagus (BE) with dysplasia is a proven risk factor for the development of esophageal adenocarcinoma.
- Observer agreement for the diagnosis of low-grade dysplasia in BE is low, prompting guidelines to advise expert review.
- This study shows that expert review can be objectified by using pre-defined benchmark quality criteria for histological assessment of BE biopsies.
- This study establishes that expertise according to benchmark criteria can be acquired and maintained using digital pathology training.
- This study implies that constant output quality within a digital pathology review panel can be maintained when expanding the number of pathologists.

## Introduction

In Barrett’s esophagus (BE), the normal stratified squamous epithelium of the distal esophagus has been replaced by columnar epithelium with or without goblet cells. Patients with BE have a risk of developing esophageal adenocarcinoma (EAC) and malignant transformation follows the metaplasia – dysplasia – carcinoma sequence.^1^ BE patients therefore undergo endoscopic surveillance. Low-grade dysplasia (LGD) in biopsies obtained during endoscopic surveillance is an accepted risk factor for progression, but diagnosis can be difficult and interobserver agreement is low.^2,3^ Therefore, guidelines advise review of all dysplastic cases by a second, preferably expert, pathologist.^4–11^

In The Netherlands we have initiated a national digital review panel for dysplastic BE, consisting of five core pathologists considered ‘experts’ in the field of BE. These five expert BE pathologists have been dedicated to the field of BE for a minimum of 10 years, have a minimum caseload of five BE cases per week of which ≥25% is dysplastic, have participated in multiple training programs (www.best-academia.eu), and have co-authored on >5 peer reviewed publications in this field. Moreover, it is the only BE expert pathologist group of individuals worldwide that have validated their BE diagnostic assessments in prospective clinical studies.^12–15^ To optimize the throughput time of the Dutch digital review panel, to divide the workload and to gain nationwide coverage, we aim to expand the panel with 10 gastrointestinal (GI) pathologists from the eight BE expert centres in The Netherlands. To maintain panel diagnostic quality we need to confirm that these pathologists’ assessments correspond with the assessment standards of the current five core pathologists. In an earlier study, we defined benchmark quality criteria, based on the assessment of the core pathologists of a study set of 60 whole-endoscopy cases.^16^ The aim of this study was to evaluate if the assessment scores of 10 dedicated GI pathologists, reviewing the same study set of 60 whole-endoscopy cases, fall within the predefined range for these benchmark quality criteria.

## Materials and methods

### Case selection and slide scanning

For 60 patients who had had an endoscopy for BE surveillance, we selected all formalin fixed, paraffin embedded tissue blocks and/or slides of the biopsies obtained during the endoscopy. The case set consisted of 39 cases with an original diagnosis of LGD (*n* = 20) or high-grade dysplasia (HGD; *n* = 19) that had been sent to our centre for consultation, between 2012 and 2014. These 39 dysplastic cases were supplemented with 21 consecutive non-dysplastic BE (NDBE) cases from a community hospital in the Amsterdam region.

The five core expert BE pathologists had assessed this case set twice individually at an earlier stage followed by consensus meetings to create a gold standard diagnosis for all cases.^16^ Their scores were used to create benchmark values for the quality criteria.

### Assessors

The assessors were 10 dedicated GI pathologists working in the eight BE expert centres in The Netherlands. In accordance with Dutch guidelines, work-up and treatment of dysplastic BE is centralized in these specialized centers. All pathologists had been dedicated to the field of BE for a median of seven years (range 5–30 years) and had a median case load of seven BE cases per week (range 5–17), of which ≥25% were dysplastic. All were actively practicing pathologists, considered experts by their peers, and had already participated in a joint training programme consisting of evaluating and discussing 60 single-slide BE cases (35 dysplastic), of which the results were published separately.^17^

### Histological assessment of samples

For the current study, the pathologists independently assessed all 60 cases twice in random order, with a wash-out time of at least one month, scoring them according to the modified Vienna criteria for GI neoplasms.^2,18^ The p53 immunohistochemistry (IHC) was used as a diagnostic adjunct and scored according to the p53 decision rule developed earlier.^17^ The pathologists individually logged onto the virtual slide system to assess the cases and record the highest diagnostic grade per case. After two assessment rounds, all cases that did not have a majority diagnosis were discussed in a face-to-face group discussion with all participants, for educational purposes.

### Definition of benchmark values for quality criteria

**Figure 1.**
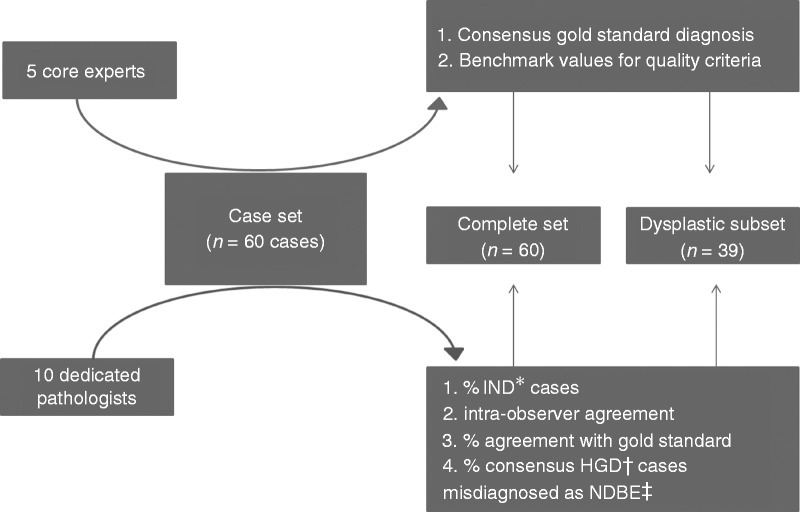
Flowchart of study set-up. *Indefinite for dysplasia; ^†^high-grade dysplasia; ^‡^non-dysplastic Barrett’s esophagus.

For each of the four quality criteria, a benchmark range had been calculated based on the scores of the five core pathologists in the aforementioned earlier study (see Table 1).^16^ The flow chart of the study can be seen in Figure 1.

**Table 1.** Values for benchmark quality criteria based on 95% prediction interval (PI) of five core pathologists.^16^

Quality criterion	95% PI core pathologists all cases (*n* = 60)	Benchmark value	95% PI core pathologists dysplastic cases (*n* = 39)	Benchmark value
Percentage of IND* cases (%)	3–14%	≤14%	−2–16%	≤16%
Intra-observer agreement in 3 categories (K)	0.66–1.02	≥0.66	0.39–0.73	≥0.39
Agreement with consensus gold standard diagnosis (%)	82–98%	≥82%	73–104%	≥73%
Consensus HGD^†^ cases misdiagnosed as NDBE^‡^ (%; fraction)	0.8% (1/120 assessments)	≤0.8% (1/120 assessments)	≤1.3% (1/78 assessments)	≤1.3% (1/78 assessments)

*IND: indefinite for dysplasia; ^†^HGD: high-grade dysplasia, ^‡^NDBE: non-dysplastic Barrett's esophagus

### Outcome measurements

**Figure 2.**
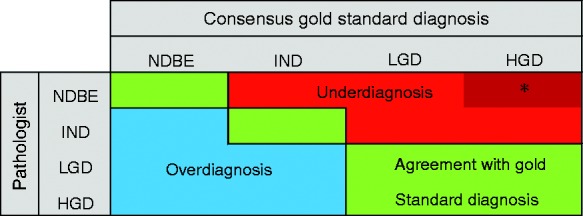
Example of 4 × 4 cross table of pathologist against consensus gold standard diagnosis, showing the position of agreement, overdiagnosis and underdiagnosis. IND: indefinite for dysplasia; LGD: low-grade dysplasia. *Significant misdiagnoses: number of consensus high-grade dysplasia (HGD) cases misdiagnosed as non-dysplastic Barrett’s oesophagus (NDBE).

Per pathologist, we established whether the scores met all benchmark quality criteria, for the complete case set as well as for the dysplastic subset (Figure 2). The different outcome measurements were calculated as per our previous study.^16^ Figure 2 illustrates the spectrum of diagnostic agreement, over- and underdiagnoses.

## Results

### Baseline characteristics of samples in case set

The median age of patients at diagnosis was 66 years (interquartile range (IQR) 13) and 73% were male. Cases contained a total of 151 sets of quadrant biopsies with a median of five slides (IQR 3–9), from a median of two levels (IQR 1–4) with four biopsies per level (IQR 3–4.5), with a total of 376 slides to be assessed.

### Performance of 10 pathologists for the complete case series (*n* = 60)

**Figure 3.**
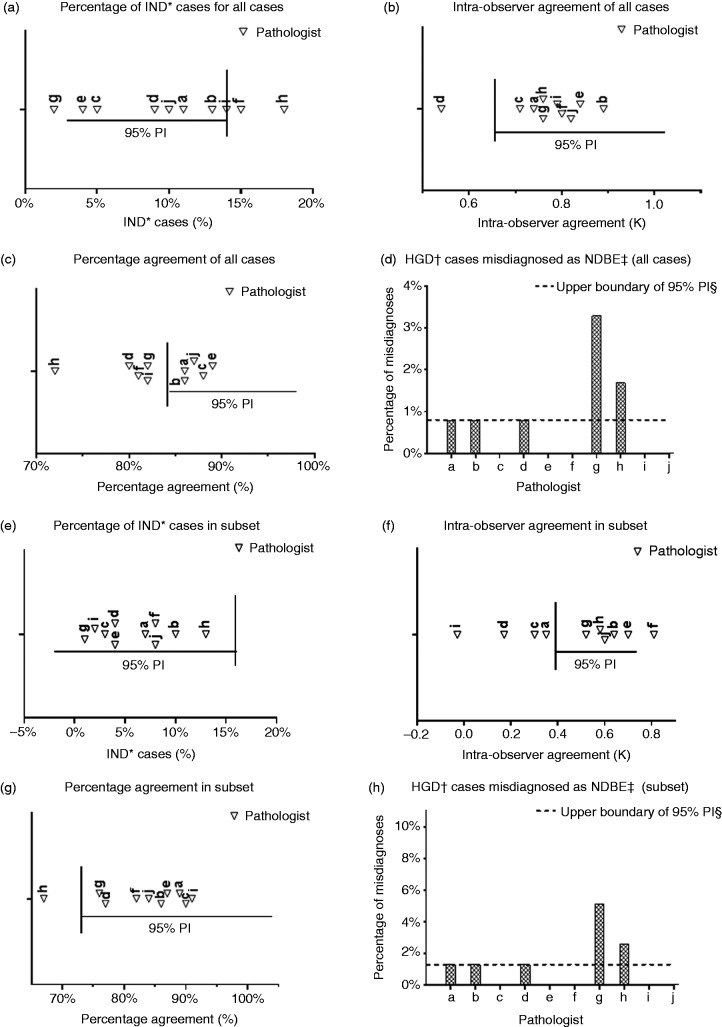
(a)–(d) Performance of 10 gastrointestinal pathologists relative to benchmark criteria, for the complete case set. (e)–(h) Performance of 10 gastrointestinal pathologists relative to benchmark criteria, for the dysplastic subset. Vertical line; benchmark value; horizontal line; 95% prediction interval. HGD: high-grade dysplasia; IND: indefinite for dysplasia; NDBE: non-dysplastic Barrett’s oesophagus. *IND: indefinite for dysplasia; ^†^HGD: high-grade dysplasia, ^‡^NDBE: non-dysplastic Barrett's esophagus, ^§^PI: prediction interval.

The pathologists generated a total of 1200 case diagnoses over 7520 assessed slides. For the percentage of indefinite for dysplasia (IND) cases, eight out of 10 pathologists met the benchmark value (see Figure 3(a)). For the intra-observer agreement, nine out of 10 pathologists fell within the benchmark value (see Figure 3(b)). For the percentage agreement with the consensus gold standard diagnosis, five out of 10 pathologists fell within the benchmark value (see Figure 3(c)). For the consensus HGD cases misdiagnosed as NDBE, eight pathologists fell within the benchmark value (see Figure 3(d)). In Supplementary Material Table 1, these results are visualised in cross tables per pathologist compared to the consensus gold standard diagnosis. For the complete case set, five out of 10 pathologists met the benchmark values for all four criteria.

### Performance of 10 pathologists for subset of dysplastic cases (*n* = 39)

For the percentage of IND cases, all pathologists fell within the benchmark value (see Figure 3(e)). For the intra-observer agreement, six out of 10 pathologists fell within the benchmark value (see Figure 3(f)). For the percentage agreement with the consensus gold standard diagnosis, nine out of 10 pathologists fell within the benchmark value (see Figure 3(g)). For the consensus HGD cases misdiagnosed as NDBE, eight out of 10 pathologists fell within the benchmark value (see Figure 3(h)). In Supplementary Material Table 2 these results are visualised in cross tables per pathologist compared to the consensus gold standard diagnosis. For the dysplastic subset, six out of 10 pathologists met the benchmark values for all four criteria.

### Performance of pathologists relative to benchmark scores

Overall, three out of 10 pathologists met all benchmark values for the complete case set as well as for the dysplastic subset. When we extended our benchmark quality criteria by using a wider range, the 99% prediction interval (PI) scores of the five core pathologists, an extra three pathologists met the benchmark range (results not shown). Four pathologists did not meet the 99% PI benchmark range on one quality criterion, namely the intra-observer agreement of the dysplastic subset, or the percentage of HGD gold standard cases misdiagnosed as NDBE.

## Discussion

The aim of this study was to test if 10 GI pathologists working at the eight BE expert centres in The Netherlands met pre-set benchmark scores of pre-defined quality criteria for evaluating BE biopsies (see Table 1), by assessing a case set of 60 BE cases consisting of 376 slides. To our knowledge, this is the first time that histopathological expertise has been quantified in the assessment of dysplastic BE biopsies. These criteria and benchmark values were established in an earlier study by using the assessments of five core expert BE pathologists as reference. These five pathologists are considered experts in BE diagnostics according to previously defined criteria: they have been dedicated to the field of BE for at least 15 years (range 15–45 years); have a median case load of seven cases per week (range 5–15), of which ≥25% are dysplastic; they participated in the Dutch Barrett advisory committee for 5–13 years^12,19,20^ and have co-authored more than 10 peer-reviewed publications in this field.^12–17,19,21–30^ To create a consensus gold standard diagnosis for this case set, these five core pathologists assessed the same dataset as used in this study, twice independently, followed by group discussions.

In the current study, the boundaries of the 95% PI and 99% PI of their individual scores were used as benchmark ranges to assess the performance of 10 dedicated GI pathologists working at the eight Dutch BE expert centers. These 10 pathologists have been dedicated to the field of BE with varying levels of experience (median of seven years (range 5–30), minimum case load of seven BE biopsies per week (range 5–17) of which ≥25% are dysplastic), however they did not have the intensive collaboration that the five core pathologists had. When comparing their assessments to the benchmark scores, we found that three out of 10 GI pathologists met all pre-set benchmark ranges for the quality criteria, for the complete case set as well as for the subset of dysplastic cases. Performance according to the benchmark range of the dysplastic subset is of key importance, since this subset is the main patient population for review requests by the national digital review panel. Their adherence to the benchmark ranges implies that these three pathologists perform similarly to the five core members in their diagnostic assessment. Expanding the digital revision panel with these three pathologists would therefore not compromise the current assessment homogeneity.

The results of our study need to be interpreted with caution. First of all, we have used intra-observer agreement (weighted kappa) as an indirect measure of expertise, because it underscores the individual reproducibility of the pathologist. However, calculating a kappa score can be less reliable when marginal totals are skewed, leading to a high chance of agreement and therefore a low kappa score. This is of particular relevance for the subanalysis of the dysplastic cases (where it is amplified by the low numbers in the subanalysis). Taking these aspects of the kappa calculation into account, we feel that the intra-observer agreement of the subanalysis is less reliable as a benchmark score than measuring diversions from the consensus gold standard diagnoses, i.e. the percentage agreement per pathologist. The percentage agreement of nine out of 10 pathologists falls within the 95% PI benchmark range for this criterion. This outcome signifies correct detection of dysplastic cases (Figure 3(g)). If we did not take the intra-observer agreement into account, one additional pathologist meets the predefined benchmark values. Second, the 95% PI benchmark range of percentage of cases ‘indefinite for dysplasia’ is inflated compared to clinical practice. This is explained by the fact that our case set was strongly enriched for difficult dysplastic cases, as encountered in a review panel setting, and by the fact that we used a p53 decision rule in the interpretation of p53 IHC as a diagnostic adjunct.^17^ Moreover, the current study is part of a structured training programme and after the individual assessments presented here, the 10 GI pathologists participated in face-to-face plenary group meetings, discussing cases that were discrepant with the consensus gold standard diagnosis. Importantly, after completion of this study set, all pathologists have assessed a case set of 62 endoscopic resection cases, and are currently reviewing 40 cases sent to the national digital review panel. These assessments were again combined with face-to-face plenary group meetings, to discuss difficult and discrepant cases. This will further improve the experience and homogeneity of panel members. We aim to reevaluate their performance in the near future, and consequently expect more pathologists to meet the benchmark quality criteria presented here.

This study has some limitations. The benchmark quality criteria used in this study depend on the distribution of diagnoses in this dataset and the individual scores of the five core pathologists. The benchmark scores only apply to this specific digital study set and the number and scores of the core pathologists. However, because there is no standardized way to define expertise in BE diagnostics, we feel that these benchmark quality criteria are currently the best choice to quantify expertise when diagnosing BE dysplasia in biopsy samples in The Netherlands.

This study is unique because of a number of features. First, it is part of a structured approach to guarantee quality and uniformity of histological diagnosis of BE biopsies in The Netherlands. Over the past five years, our group has set up a national digital review panel for BE after conducting five preliminary studies.^16,17,30^ This is the first time worldwide that an expert pathology review panel has been set up conducting such quantifiable preliminary work in such a meticulous way. Second, the case set used for this study consisted of all slides from all biopsy levels of a single endoscopy (376 slides in total), was fully digitalized and only contained review cases from clinical practice. There were two assessment rounds with an adequate wash-out time. In order to improve homogeneity of the group the pathologists held a group discussion afterwards to discuss cases that did not have a majority diagnosis. This digital case set of dysplastic BE cases will be made available to allow pathologists in and outside The Netherlands to evaluate if they meet the aforementioned benchmark ranges for quality criteria.

Our goal for the future remains to improve the knowledge of BE-related diagnostic pathology among GI pathologists in The Netherlands; and to include all GI pathologists working at the BE expert centers in The Netherlands in our review panel. For this, we first need to ensure quality and homogeneity of the panel as outlined above. Subsequently, we need a prediction model that allows us to efficiently select the number of pathologists needed for reviewing cases and to divide the workload equally among panel members. We aim to improve and expand training in BE pathology both nationally as well as internationally by constructing a freely available, accredited training module incorporating the information gathered from all study sets and group discussions thus far.^16,17,30^ In this way, pathologists with an interest in BE can train themselves and reflect on their performance relative to the benchmark scores of the training set.

## Supplemental Material

Supplemental material for Adherence to pre-set benchmark quality criteria to qualify as expert assessor of dysplasia in Barrett’s esophagus biopsies – towards digital review of Barrett’s esophagus Click here for additional data file. Supplemental Material for Adherence to pre-set benchmark quality criteria to qualify as expert assessor of dysplasia in Barrett’s esophagus biopsies – towards digital review of Barrett’s esophagus by MJ van der Wel, E Klaver, LC Duits, RE Pouw, CA Seldenrijk, GJA Offerhaus, M Visser, FJW ten Kate, K Biermann, LAA Brosens, M Doukas, C Huysentruyt, A Karrenbeld, G Kats-Ugurlu, JS van der Laan, G van Lijnschoten, FCP Moll, AHAG Ooms, JG Tijssen, JJGHM Bergman and SL Meijer in United European Gastroenterology Journal

## Research Data

Research Data for Adherence to pre-set benchmark quality criteria to qualify as expert assessor of dysplasia in Barrett’s esophagus biopsies – towards digital review of Barrett’s esophagus Click here for additional data file. Research Data for Adherence to pre-set benchmark quality criteria to qualify as expert assessor of dysplasia in Barrett’s esophagus biopsies – towards digital review of Barrett’s esophagus by MJ van der Wel, E Klaver, LC Duits, RE Pouw, CA Seldenrijk, GJA Offerhaus, M Visser, FJW ten Kate, K Biermann, LAA Brosens, M Doukas, C Huysentruyt, A Karrenbeld, G Kats-Ugurlu, JS van der Laan, G van Lijnschoten, FCP Moll, AHAG Ooms, JG Tijssen, JJGHM Bergman and SL Meijer in United European Gastroenterology Journal This article is distributed under the terms of the Creative
Commons Attribution 4.0 License ( http://www.creativecommons.org/licenses/by/4.0/) which
permits any use, reproduction and distribution of the work without
further permission provided the original work is attributed as specified
on the SAGE and Open Access pages ( https://us.sagepub.com/en-us/nam/open-access-at-sage).

## References

[1] Haggitt RC Tryzelaar J Ellis FH, et al. Adenocarcinoma complicating columnar epithelium-lined (Barrett's) esophagus. Am J Clin Pathol 1978; 70: 1–5. 696666 10.1093/ajcp/70.1.1

[2] Reid BJ Haggitt RC Rubin CE, et al. Observer variation in the diagnosis of dysplasia in Barrett's esophagus. Hum Pathol 1988; 19: 166–178. 3343032 10.1016/s0046-8177(88)80344-7

[3] Montgomery E Bronner MP Goldblum JR, et al. Reproducibility of the diagnosis of dysplasia in Barrett esophagus: A reaffirmation. Hum Pathol 2001; 32: 368–378. 11331953 10.1053/hupa.2001.23510

[4] Fitzgerald RC di Pietro M Ragunath K, et al. British Society of Gastroenterology guidelines on the diagnosis and management of Barrett's oesophagus. Gut 2014; 63: 7–42. 24165758 10.1136/gutjnl-2013-305372

[5] American Gastroenterological A Spechler SJ Sharma P, et al. American Gastroenterological Association medical position statement on the management of Barrett's esophagus. Gastroenterology 2011; 140: 1084–1091. 21376940 10.1053/j.gastro.2011.01.030

[6] Fock KM Talley N Goh KL, et al. Asia-Pacific consensus on the management of gastro-oesophageal reflux disease: An update focusing on refractory reflux disease and Barrett's oesophagus. Gut 2016; 65: 1402–1415. 27261337 10.1136/gutjnl-2016-311715

[7] Shaheen NJ Falk GW Iyer PG, et al. ACG clinical guideline: Diagnosis and management of Barrett's esophagus. Am J Gastroenterol 2016; 111: 30–50. 26526079 10.1038/ajg.2015.322 PMC10245082

[8] Wang KK Sampliner RE Practice Parameters Committee of the American College of Gastroenterology Updated guidelines 2008 for the diagnosis, surveillance and therapy of Barrett's esophagus. Am J Gastroenterol 2008; 103: 788–797. 18341497 10.1111/j.1572-0241.2008.01835.x

[9] Weusten B Bisschops R Coron E, et al. Endoscopic management of Barrett's esophagus: European Society of Gastrointestinal Endoscopy (ESGE) position statement. Endoscopy 2017; 49: 191–198. 28122386 10.1055/s-0042-122140

[10] Whiteman DC, Appleyard M, Bahin FF, et al. Australian clinical practice guidelines for the diagnosis and management of Barrett's esophagus and early esophageal adenocarcinoma. *J Gastroenterol Hepatol* 2015; 30: 804–820. doi: 10.1111/jgh.12913. 10.1111/jgh.12913 25612140

[11] Richtlijn Barrett-oesofagus [in Dutch]: https://www.mdl.nl/sites/www.mdl.nl/files/richlijnen/Richtlijnen%20Barrett%20oesofagus%20-%20jan%202018%20-%20tbv%20website.pdf.

[12] Duits LC, Phoa KN, Curvers WL, et al. Barrett's oesophagus patients with low-grade dysplasia can be accurately risk-stratified after histological review by an expert pathology panel. *Gut* 2015; 64: 700–706. doi: 10.1136/gutjnl-2014-307278. Epub 17 July 2014. 10.1136/gutjnl-2014-307278 25034523

[13] Duits LC van der Wel MJ Cotton CC, et al. Patients with Barrett's esophagus and confirmed persistent low-grade dysplasia are at increased risk for progression to neoplasia. Gastroenterology 2017; 152: 993–1001. 28012849 10.1053/j.gastro.2016.12.008

[14] Phoa KN van Vilsteren FG Weusten BL, et al. Radiofrequency ablation vs endoscopic surveillance for patients with Barrett esophagus and low-grade dysplasia: A randomized clinical trial. JAMA 2014; 311: 1209–1217. 24668102 10.1001/jama.2014.2511

[15] Curvers WL ten Kate FJ Krishnadath KK, et al. Low-grade dysplasia in Barrett's esophagus: Overdiagnosed and underestimated. Am J Gastroenterol 2010; 105: 1523–1530. 20461069 10.1038/ajg.2010.171

[16] van der Wel MJ Duits LC Klaver E, et al. Development of benchmark quality criteria for assessing whole-endoscopy Barrett's esophagus biopsy cases. United European Gastroenterol J 2018; 6: 830–837. 10.1177/2050640618764710 PMC6047285 30023060

[17] van der Wel MJ Duits LC Pouw RE, et al. Improved diagnostic stratification of digitised Barrett's oesophagus biopsies by TP53 immunohistochemical staining. Histopathology 2018; 72: 1015–1023. 29314176 10.1111/his.13462

[18] Schlemper R Riddell R Kato Y, et al. The Vienna classification of gastrointestinal epithelial neoplasia. Gut 2000; 47: 251–255. 10896917 10.1136/gut.47.2.251 PMC1728018

[19] Offerhaus GJ Correa P van Eeden S, et al. Report of an Amsterdam working group on Barrett esophagus. Virchows Arch 2003; 443: 602–608. 14517678 10.1007/s00428-003-0906-z

[20] Hulscher JB Haringsma J Benraadt J, et al. Comprehensive Cancer Centre Amsterdam Barrett Advisory Committee: First results. Neth J Med 2001; 58: 3–8. 11137744 10.1016/s0300-2977(00)00086-3

[21] Curvers WL van Vilsteren FG Baak LC, et al. Endoscopic trimodal imaging versus standard video endoscopy for detection of early Barrett's neoplasia: A multicenter, randomized, crossover study in general practice. Gastrointest Endosc 2011; 73: 195–203. 21168835 10.1016/j.gie.2010.10.014

[22] Polkowski W Baak JP van Lanschot JJ, et al. Clinical decision making in Barrett's oesophagus can be supported by computerized immunoquantitation and morphometry of features associated with proliferation and differentiation. J Pathol 1998; 184: 161–168. 9602707 10.1002/(SICI)1096-9896(199802)184:2<161::AID-PATH971>3.0.CO;2-2

[23] van Sandick JW Baak JP van Lanschot JJ, et al. Computerized quantitative pathology for the grading of dysplasia in surveillance biopsies of Barrett's oesophagus. J Pathol 2000; 190: 177–183. 10657016 10.1002/(SICI)1096-9896(200002)190:2<177::AID-PATH508>3.0.CO;2-X

[24] van Sandick JW van Lanschot JJ Kuiken BW, et al. Impact of endoscopic biopsy surveillance of Barrett's oesophagus on pathological stage and clinical outcome of Barrett's carcinoma. Gut 1998; 43: 216–222. 10189847 10.1136/gut.43.2.216 PMC1727211

[25] Phoa KN, Pouw RE, Bisschops R, et al. Multimodality endoscopic eradication for neoplastic Barrett oesophagus: Results of an European multicentre study (EURO-II). *Gut* 2016; 65: 555–562. doi: 10.1136/gutjnl-2015-309298. Epub 2 March 2015. 10.1136/gutjnl-2015-309298 25731874

[26] Alvarez Herrero L van Vilsteren FG Pouw RE, et al. Endoscopic radiofrequency ablation combined with endoscopic resection for early neoplasia in Barrett's esophagus longer than 10 cm. Gastrointest Endosc 2011; 73: 682–690. 21292262 10.1016/j.gie.2010.11.016

[27] van Vilsteren FG Pouw RE Seewald S, et al. Stepwise radical endoscopic resection versus radiofrequency ablation for Barrett's oesophagus with high-grade dysplasia or early cancer: A multicentre randomised trial. Gut 2011; 60: 765–773. 21209124 10.1136/gut.2010.229310

[28] Phoa KN Pouw RE van Vilsteren FG, et al. Remission of Barrett's esophagus with early neoplasia 5 years after radiofrequency ablation with endoscopic resection: A Netherlands cohort study. Gastroenterology 2013; 145: 96–104. 23542068 10.1053/j.gastro.2013.03.046

[29] Peters FP Brakenhoff KP Curvers WL, et al. Histologic evaluation of resection specimens obtained at 293 endoscopic resections in Barrett's esophagus. Gastrointest Endosc 2008; 67: 604–609. 18155214 10.1016/j.gie.2007.08.039

[30] van der Wel MJ Duits LC Seldenrijk CA, et al. Digital microscopy as valid alternative to conventional microscopy for histological evaluation of Barrett's esophagus biopsies. Dis Esophagus 2017; 30: 1–7. 10.1093/dote/dox078 28881901

