# Supplementary material for: Adherence to pre-set benchmark quality criteria to qualify as expert assessor of dysplasia in Barrett’s esophagus biopsies – towards digital review of Barrett’s esophagus
Source: United European Gastroenterol J. 2019 May 21;7(7):889–96. doi: 10.1177/2050640619853441 (PMC6683647; doi:10.1177/2050640619853441)
Supplement: Supplemental material for Adherence to pre-set benchmark quality criteria to qualify as expert assessor of dysplasia in Barrett’s esophagus biopsies – towards digital review of Barrett’s esophagus [file Supplemental1_Material.pdf]

Supplementary table 1: Agreement of 10 GI* pathologists with consensus gold standard diagnosis (mean over 2 assessment rounds) for the complete case set (n=60) in 4 categories (4x4 cross tables)

	Observed						Observed (%)					
			GS						GS			
		NDBE	IND	LGD	HGD	Total		NDBE	IND	LGD	HGD	Total
	NDBE	17.0	1.0	2.5	0.5	21.0		28.4%	1.7%	4.2%	0.9%	35.0%
P1	IND	1.0	2.0	2.5	1.0	6.5		1.7%	3.3%	4.2%	1.7%	10.9%
	LGD	0.0	0.0	9.5	5.0	14.5		0.0%	0.0%	15.9%	8.4%	24.2%
	HGD	0.0	0.0	4.5	13.5	18.0		0.0%	0.0%	7.5%	22.5%	30.0%
	Total	18.0	3.0	19.0	20.0	60.0		30.0%	5.0%	31.7%	33.3%	100.0%
	Observed						Observed (%)					
			GS						GS			
		NDBE	IND	LGD	HGD	Total		NDBE	IND	LGD	HGD	Total
	NDBE	18.0	1.5	0.0	0.5	20.0		30.0%	2.5%	0.0%	0.9%	33.3%
P2	IND	0.0	1.5	6.5	0.0	8.0		0.0%	2.5%	10.9%	0.0%	13.4%
	LGD	0.0	0.0	11.0	3.5	14.5		0.0%	0.0%	18.3%	5.9%	24.2%
	HGD	0.0	0.0	1.5	16.0	17.5		0.0%	0.0%	2.5%	26.7%	29.2%
	Total	18.0	3.0	19.0	20.0	60.0		30.0%	5.0%	31.7%	33.3%	100.0%
	Observed						Observed (%)					
			GS						GS			
		NDBE	IND	LGD	HGD	Total		NDBE	IND	LGD	HGD	Total
	NDBE	15.0	0.5	0.0	0.0	15.5		25.0%	1%	0.0%	0.0%	25.8%
P3	IND	1.0	0.5	1.0	0.5	3.0		1.7%	0.8%	1.7%	0.8%	5.0%
	LGD	1.5	2.0	16.0	5.0	24.5		2.5%	3.3%	26.7%	8.3%	40.8%
	HGD	0.5	0.0	2.0	14.5	17.0		0.9%	0.0%	3.4%	24.2%	28.3%
	Total	18.0	3.0	19.0	20.0	60.0		30.0%	5.0%	31.7%	33.3%	100.0%
	Observed						Observed (%)					
			GS						GS			
		NDBE	IND	LGD	HGD	Total		NDBE	IND	LGD	HGD	Total
	NDBE	16.5	1.0	4.0	0.5	22.0		27.5%	1.7%	6.7%	0.9%	36.7%
P4	IND	1.5	0.5	3.0	0.5	5.5		2.5%	0.9%	5.0%	0.9%	9.2%
	LGD	0.0	1.5	10.5	8.5	20.5		0.0%	2.5%	17.5%	14.2%	34.2%
	HGD	0.0	0.0	1.5	10.5	12.0		0.0%	0.0%	2.5%	17.5%	20.0%
	Total	18.0	3.0	19.0	20.0	60.0		30.0%	5.0%	31.7%	33.3%	100.0%
	Observed						Observed (%)					
			GS						GS			
		NDBE	IND	LGD	HGD	Total		NDBE	IND	LGD	HGD	Total
	NDBE	18.0	2.5	1.0	0.0	21.5		30.0%	4.2%	1.7%	0.0%	35.9%
P5	IND	0.0	0.0	2.0	0.5	2.5		0.0%	0.0%	3.4%	0.9%	4.2%
	LGD	0.0	0.5	13.5	9.5	23.5		0.0%	0.9%	22.5%	15.8%	39.2%
	HGD	0.0	0.0	2.	10.0	12.5		0.0%	0.0%	4.2%	16.7%	20.8%
	Total	18.0	3.0	19.0	20.0	60.0		30.0%	5.0%	31.7%	33.3%	100.0%
	Observed						Observed (%)					
			GS						GS			
		NDBE	IND	LGD	HGD	Total		NDBE	IND	LGD	HGD	Total
	NDBE	13.5	0.0	0.0	0.0	13.5		22.5%	0.0%	0.0%	0.0%	22.5%
P6	IND	4.0	0.5	4.0	0.5	9.0		6.7%	0.9%	6.7%	0.9%	15.0%
	LGD	0.5	2.5	14.5	6.0	23.5		0.9%	4.2%	24.2%	10.0%	39.2%
	HGD	0.0	0.0	0.5	13.5	14.0		0.0%	0.0%	0.9%	22.5%	23.4%
	Total	18.0	3.0	19.0	20.0	60.0		30.0%	5.0%	31.7%	33.3%	100.0%
	Observed						Observed (%)					
			GS						GS			
		NDBE	IND	LGD	HGD	Total		NDBE	IND	LGD	HGD	Total
	NDBE	17.0	2.0	5.0	2.0	26.0		28.4%	3.3%	8.4%	3.3%	43.3%
P7	IND	1.0	0.0	0.0	0.0	1.0		1.7%	0.0%	0.0%	0.0%	1.7%
	LGD	0.0	1.0	13.5	13.5	28.0		0.0%	1.7%	22.5%	22.5%	46.7%
	HGD	0.0	0.0	0.5	4.5	5.0		0.0%	0.0%	0.9%	7.5%	8.3%
	Total	18.0	3.0	19.0	20.0	60.0		30.0%	5.0%	31.7%	33.3%	100.0%
	Observed						Observed (%)					
			GS						GS			
		NDBE	IND	LGD	HGD	Total		NDBE	IND	LGD	HGD	Total

	NDBE	16.5	2.0	3.0	1.0	22.5		27.5%	3.3%	5.0%	1.7%	37.5%
P8	IND	1.5	0.5	7.5	1.5	11.0		2.5%	0.9%	12.5%	2.5%	18.0%
	LGD	0.0	0.5	5.5	7.0	13.0		0.0%	0.9%	9.2%	11.7%	21.7%
	HGD	0.0	0.0	3.0	10.5	13.5		0.0%	0.0%	5.0%	17.5%	22.5%
	Total	18.0	3.0	19.0	20.0	60.0		30.0%	5.0%	31.7%	33.3%	100.0%
	Observed						Observed (%)					
			GS						GS			
		NDBE	IND	LGD	HGD	Total		NDBE	IND	LGD	HGD	Total
	NDBE	10.0	0.0	0.0	0.0	10.0		16.7%	0.0%	0.0%	0.0%	16.7%
P9	IND	6.5	1.0	1.0	0.0	8.5		10.9%	1.7%	1.7%	0.0%	14.2%
	LGD	1.5	2.0	15.0	3.5	22.0		2.5%	3.3%	25.0%	5.8%	36.7%
	HGD	0.0	0.0	3.0	16.5	19.5		0.0%	0.0%	5.0%	27.5%	32.5%
	Total	18.0	3.0	19.0	20.0	60.0		30.0%	5.0%	31.7%	33.3%	100.0%
	Observed						Observed (%)					
			GS						GS			
		NDBE	IND	LGD	HGD	Total		NDBE	IND	LGD	HGD	Total
	NDBE	17.5	1.5	1.0	0.0	20.0		29.2%	2.5%	1.7%	0.0%	33.3%
P10	IND	0.5	1.0	4.5	0.0	6.0		0.9%	1.7%	7.5%	0.0%	10.0%
	LGD	0.0	0.5	11.0	3.0	14.5		0.0%	0.9%	18.3%	5.0%	24.2%
	HGD	0.0	0.0	2.5	17.0	19.5		0.0%	0.0%	4.2%	28.3%	32.5%
	Total	18.0	3.0	19.0	20.0	60.0		30.0%	5.0%	31.7%	33.3%	100.0%

*GI = gastrointestinal; **NDBE = non-dysplastic Barrett's oesophagus; ***IND = indefinite for dysplasia; ****LGD = low-grade dysplasia; *****HGD = high-grade dysplasia

Supplementary table 2: Agreement of 10 GI* pathologists with consensus gold standard diagnosis (mean over 2 assessment rounds) for the dysplastic subset (n=39) in 4 categories (4x4 cross tables)

	Observed						Observed (%)					
			GS						GS			
		NDBE	IND	LGD	HGD	Total		NDBE	IND	LGD	HGD	Total
	NDBE	1.0	0.0	2.0	0.5	3.5		2.6%	0.0%	5.1%	1.3%	9.0%
P1	IND	0.0	2.0	1.0	1.0	4.0		0.0%	5.1%	2.6%	2.6%	10.3%
	LGD	0.0	0.0	9.5	5.0	14.5		0.0%	0.0%	24.4%	12.8%	37.2%
	HGD	0.0	0.0	3.5	13.5	17.0		0.0%	0.0%	9.0%	34.7%	43.6%
	Total	1.0	2.0	16.0	20.0	39.0		2.6%	5.1%	41.0%	51.3%	100.0%
	Observed						Observed (%)					
			GS						GS			
		NDBE	IND	LGD	HGD	Total		NDBE	IND	LGD	HGD	Total
	NDBE	1.0	0.5	0.0	0.5	2.0		2.6%	1.3%	0.0%	1.3%	5.1%
P2	IND	0.0	1.5	4.5	0.0	6.0		0.0%	3.9%	11.6%	0.0%	15.4%
	LGD	0.0	0.0	10.0	3.5	13.5		0.0%	0.0%	25.6%	9.0%	34.6%
	HGD	0.0	0.0	1.5	16.0	17.5		0.0%	0.0%	3.9%	41.0%	44.9%
	Total	1.0	2.0	16.0	20.0	39.0		2.6%	5.1%	41.0%	51.3%	100.0%
	Observed						Observed (%)					
			GS						GS			
		NDBE	IND	LGD	HGD	Total		NDBE	IND	LGD	HGD	Total
	NDBE	0.5	0.0	0.0	0.0	0.5		1.3%	0.0%	0.0%	0.0%	1.3%
P3	IND	0.5	0.0	1.0	0.5	2.0		1.3%	0.0%	2.6%	1.3%	5.1%
	LGD	0.0	2.0	13.5	5.0	20.5		0.0%	5.1%	34.7%	12.8%	52.6%
	HGD	0.0	0.0	1.5	14.5	16.0		0.0%	0.0%	3.9%	37.2%	41.1%
	Total	1.0	2.0	16.0	20.0	39.0		2.6%	5.1%	41.0%	51.3%	100.0%
	Observed						Observed (%)					
			GS						GS			
		NDBE	IND	LGD	HGD	Total		NDBE	IND	LGD	HGD	Total
	NDBE	1.0	0.5	4.0	0.5	6.0		2.6%	1.3%	10.3%	1.3%	15.4%
P4	IND	0.0	0.0	2.0	0.5	2.5		0.0%	0.0%	5.2%	1.3%	6.4%
	LGD	0.0	1.5	8.5	8.5	18.5		0.0%	3.9%	21.8%	21.8%	47.4%
	HGD	0.0	0.0	1.5	10.5	12.0		0.0%	0.0%	3.9%	27.0%	30.8%
	Total	1.0	2.0	16.0	20.0	39.0		2.6%	5.1%	41.0%	51.3%	100.0%
	Observed						Observed (%)					
			GS						GS			
		NDBE	IND	LGD	HGD	Total		NDBE	IND	LGD	HGD	Total
	NDBE	1.0	2.0	0.5	0.0	3.5		2.6%	5%	1.3%	0.0%	9.0%
P5	IND	0.0	0.0	2.0	0.5	2.5		0.0%	0.0%	5.2%	1.3%	6.4%
	LGD	0.0	0.0	11.0	9.5	20.5		0.0%	0.0%	28.2%	24.4%	52.6%
	HGD	0.0	0.0	2.5	10.0	12.5		0.0%	0.0%	6.4%	25.7%	32.1%
	Total	1.0	2.0	16.0	20.0	39.0		2.6%	5.1%	41.0%	51.3%	100.0%
	Observed						Observed (%)					
			GS						GS			
		NDBE	IND	LGD	HGD	Total		NDBE	IND	LGD	HGD	Total
	NDBE	0.5	0.0	0.0	0.0	0.5		1.3%	0.0%	0.0%	0.0%	1.3%
P6	IND	0.5	0.0	4.0	0.5	5.0		1.3%	0.0%	10.3%	1.3%	12.9%
	LGD	0.0	2.0	11.5	6.0	19.5		0.0%	5.1%	29.5%	15.4%	50.0%
	HGD	0.0	0.0	0.5	13.5	14.0		0.0%	0.0%	1.3%	34.6%	35.9%
	Total	1.0	2.0	16.0	20.0	39.0		2.6%	5.1%	41.0%	51.3%	100.0%
	Observed						Observed (%)					
			GS						GS			
		NDBE	IND	LGD	HGD	Total		NDBE	IND	LGD	HGD	Total
	NDBE	0.5	1.0	5.0	2.0	8.5		1.3%	2.6%	12.9%	5.1%	21.8%
P7	IND	0.5	0.0	0.0	0.0	0.5		1.3%	0.0%	0.0%	0.0%	1.3%
	LGD	0.0	1.0	10.5	13.5	25.0		0.0%	2.6%	26.9%	34.6%	64.1%
	HGD	0.0	0.0	0.5	4.5	5.0		0.0%	0.0%	1.3%	11.6%	12.8%
	Total	1.0	2.0	16.0	20.0	39.0		2.6%	5.1%	41.0%	51.3%	100.0%
	Observed						Observed (%)					
			GS						GS			
		NDBE	IND	LGD	HGD	Total		NDBE	IND	LGD	HGD	Total

	NDBE	1.0	1.0	3.0	1.0	6.0		2.6%	2.6%	7.7%	2.6%	15.4%
P8	IND	0.0	0.5	6.0	1.5	8.0		0.0%	1.3%	15.4%	3.9%	20.5%
	LGD	0.0	0.5	5.0	7.0	12.5		0.0%	1.3%	12.8%	18.0%	32.1%
	HGD	0.0	0.0	2.0	10.5	12.5		0.0%	0.0%	5.1%	26.9%	32.1%
	Total	1.0	2.0	16.0	20.0	39.0		2.6%	5.1%	41.0%	51.3%	100.0%
	Observed						Observed (%)					
			GS						GS			
		NDBE	IND	LGD	HGD	Total		NDBE	IND	LGD	HGD	Total
	NDBE	0.5	0.0	0.0	0.0	0.5		1.3%	0.0%	0.0%	0.0%	1.3%
P9	IND	0.0	0.0	1.0	0.0	1.0		0.0%	0.0%	2.6%	0.0%	2.6%
	LGD	0.5	2.0	12.5	3.5	18.5		1.3%	5.1%	32.1%	9.0%	47.5%
	HGD	0.0	0.0	2.5	16.5	19.0		0.0%	0.0%	6.5%	42.4%	48.7%
	Total	1.0	2.0	16.0	20.0	39.0		2.6%	5.1%	41.0%	51.3%	100.0%
	Observed						Observed (%)					
			GS						GS			
		NDBE	IND	LGD	HGD	Total		NDBE	IND	LGD	HGD	Total
	NDBE	1.0	1.0	1.0	0.0	0.5		2.6%	2.6%	2.6%	0.0%	7.7%
P10	IND	0.0	0.5	4.0	0.0	1.0		0.0%	1.3%	10.3%	0.0%	11.6%
	LGD	0.0	0.5	9.0	3.0	18.5		0.0%	1.3%	23.1%	7.7%	32.1%
	HGD	0.0	0.0	2.0	17.0	19.0		0.0%	0.0%	5.2%	43.6%	48.8%
	Total	1.0	2.0	16.0	20.0	39.0		2.6%	5.1%	41.0%	51.3%	100.0%

*GI = gastrointestinal; **NDBE = non-dysplastic Barrett's oesophagus; ***IND = indefinite for dysplasia; ****LGD = low-grade dysplasia; *****HGD = high-grade dysplasia
