# Supplementary material for: Adherence to pre-set benchmark quality criteria to qualify as expert assessor of dysplasia in Barrett’s esophagus biopsies – towards digital review of Barrett’s esophagus
Source: United European Gastroenterol J. 2019 May 21;7(7):889–96. doi: 10.1177/2050640619853441 (PMC6683647; doi:10.1177/2050640619853441)
Supplement: Research Data for Adherence to pre-set benchmark quality criteria to qualify as expert assessor of dysplasia in Barrett’s esophagus biopsies – towards digital review of Barrett’s esophagus [file Research_Data.pdf]

Amsterdam UMC
Universitair Medische Centra

Aan de heer prof.dr. J.J.G.H.M. Bergman
Maag-, Darm- Leverziekten
C2-113

Medisch Ethische Toetsingscommissie AMC
XT4-148
telefoon: 020 56 67389

Amsterdam, 14 februari 2019
uw kenmerk:
ons kenmerk: W19_050# 19.072
betreft:
Uw project: **Adherence to pre-set benchmark quality criteria to qualify as expert assessor of dysplasia in Barrett's oesophagus biopsies**

Geachte heer Bergman

Uw brief d.d. 1 februari 2019 betreffende bovengenoemde studie is op 13 februari jl. besproken in de vergadering van het dagelijks bestuur.

Het dagelijks bestuur is van oordeel dat bovengenoemde studie niet valt binnen de reikwijdte van de Wet medisch-wetenschappelijk onderzoek met mensen, aangezien er geen sprake is van wetenschappelijk onderzoek zoals bedoeld in artikel 1, eerste lid onder b van de WMO, daar er geen proefpersonen zijn alleen geanonimiseerd pathologisch materiaal.

Een formele beoordeling door onze commissie is derhalve niet noodzakelijk.

De commissie attendeert u op de volgende punten:
De commissie heeft alleen de WMO-plichtigheid beoordeeld. Er heeft verder geen inhoudelijke toets van het onderzoek plaatsgevonden. U en uw afdeling zijn verantwoordelijk voor de correcte uitvoering van het onderzoek volgens de geldende wet- en regelgeving. Hierbij vragen wij uw aandacht voor de belangrijkste regelgeving:

- Voor prospectief onderzoek, waarbij gegevens van proefpersonen worden verzameld en verwerkt, is toestemming van de proefpersonen nodig.
- Voor retrospectief onderzoek, waarbij gegevens van proefpersonen gecodeerd worden verzameld en verwerkt is in beginsel toestemming van de proefpersonen nodig. In artikel 458 van de WGBO is vastgelegd onder welke omstandigheden van het vragen van toestemming kan worden afgezien. Bij retrospectief *anoniem* onderzoek is toestemming niet verplicht, hierbij zijn de gegevens nooit meer herleidbaar tot de proefpersonen. Dus ook niet via een code.
- Wanneer in een onderzoek gegevens worden verzameld van proefpersonen, dient hiermee correct te worden omgegaan zoals bepaald in de Gedragscode Gezondheidsonderzoek (Code Goed Gedrag), Algemene Verordening Gegevensbescherming en de Uitvoeringswet Algemene Verordening Gegevensbescherming en, indien het onderzoek van het AMC betreft, de regels die binnen het AMC zijn vastgesteld, zoals de SOP "Reuse of care data for the purpose of research van de CRU (http:/frntran et.amc.nl/web/organisatie/domeinen/research/clinical-research-unitu/cru-home/sops-dm.htm)
- Wanneer in een onderzoek (lichaams)materiaal van proefpersonen wordt verzameld en verwerkt dient hiermee correct te worden omgegaan zoals bepaald in de Code Goed Gebruik. Indien er sprake is van een biobank in het AMC, dat wil zeggen dat het lichaamsmateriaal in het AMC wordt opgeslagen met het oog op toekomstig onderzoek, dient dit te worden voorgelegd aan de BiobankToetsingsCommissie (BTC) van het AMC.
- Onderzoek met anoniem materiaal vanuit de zorg is toegestaan, voorzover de patiënt van wie het materiaal afkomstig is hier geen bezwaar tegen heeft gemaakt (artikel 467 WGBO).
- Voorts dient u zich te houden aan de research code van het AMC en het VUmc.

Meer informatie over bovengenoemde regelgeving kunt u vinden op internet, waaronder onze intranetpagina.
Deze opsomming betreft de belangrijkste regelgeving, maar is niet uitputtend. Mogelijk is nog

andere wet- en regelgeving van toepassing op uw onderzoek.

Indien u twijfelt of door amendering of het toevoegen van addenda het onderzoek nog steeds buiten de reikwijdte van de WMO blijft kunt u dit aan de commissie ter beoordeling voorleggen.

Met vriendelijke groet,
namens de Medisch Ethische Toetsingscommissie AMC,

Mw. dr. C.L. van der Wilt
ambtelijk secretaris

Bijlage: verklaring in het Engels

e.c. per email: m.j.vanderwel@amc.uva.nl; s.l.meijer@amc.uva.nl

To whom it may concern,

Referring to our letter of February, 14, 2019 (reference number W19_050 # 19.072) we are pleased to confirm that the Medical Research Involving Human Subjects Act (WMO) does not apply to the above mentioned study and that an official approval of this study by our committee is not required.

Yours sincerely,
on behalf of the Medical Ethics Review Committee of the Academie Medical Center,

Mrs. C.L. van der Wilt, PhD
secretary

AMC en VUmc werken samen in Amsterdam UMC

AMC
Meibergdreef 9
1105 AZ Amsterdam

Postbus 22660
1100 DD Amsterdam

T +31(0)20 566 9111
www.amc.nl
